# Optimization of a parallel CAR for B-cell lymphoma via ITAM attenuation and target specificity validation

**DOI:** 10.1093/cei/uxag032

**Published:** 2026-05-26

**Authors:** Fahima Kausar, Christopher Davis, Daniel Larcombe-Young, Camilla Bove, Farzin Farzaneh, Charlotte Graham, Reuben Benjamin, David M Davies, John Maher

**Affiliations:** Leucid Bio Ltd., Guy’s Hospital, Great Maze Pond, London SE1 9RT, UK; Leucid Bio Ltd., Guy’s Hospital, Great Maze Pond, London SE1 9RT, UK; Leucid Bio Ltd., Guy’s Hospital, Great Maze Pond, London SE1 9RT, UK; King’s College London, CAR Mechanics Group, Department of Precision & Population Oncology, School of Cancer and Pharmaceutical Sciences, London SE1 9RT, UK; Leucid Bio Ltd., Guy’s Hospital, Great Maze Pond, London SE1 9RT, UK; King’s College London, Department of Haematology, School of Cancer and Pharmaceutical Sciences, London SE5 9RS, UK; ViroCell Biologics, Zayed Centre for Research into Rare Diseases in Children, London WC1N 1DZ, UK; King’s College London, Department of Haematology, School of Cancer and Pharmaceutical Sciences, London SE5 9RS, UK; King’s College Hospital, Department of Haematology, London SE5 9RS, UK; King’s College London, Department of Haematology, School of Cancer and Pharmaceutical Sciences, London SE5 9RS, UK; King’s College Hospital, Department of Haematology, London SE5 9RS, UK; Leucid Bio Ltd., Guy’s Hospital, Great Maze Pond, London SE1 9RT, UK; Leucid Bio Ltd., Guy’s Hospital, Great Maze Pond, London SE1 9RT, UK; King’s College London, CAR Mechanics Group, Department of Precision & Population Oncology, School of Cancer and Pharmaceutical Sciences, London SE1 9RT, UK; Department of Immunology, Eastbourne Hospital, Kings Drive, Eastbourne, East Sussex BN21 2UD, UK

**Keywords:** parallel chimeric antigen receptor, B-cell, CD28, 4-1BB, ITAM, 1XX

## Abstract

Second-generation CAR T-cells have transformed the management of B-cell malignancy. However, *in vivo* functional persistence is often limited, highlighting a key mechanism of treatment failure. We have engineered a parallel (p)CAR platform that delivers dual CD28 and 4-1BB co-stimulation via a co-expressed CAR and chimeric co-stimulatory receptor (CCR). To target CD19, we employed an avidity-optimized FMC63 scFv to direct CAR specificity while utilizing an unmodified FMC63 scFv to target the CCR. Here, we describe the late-stage optimization of this system for Phase 1 clinical evaluation. First, we confirmed that the avidity-optimized scFv lacked off-target specificity. To minimize risk of insertional mutagenesis and immunogenicity, respectively, we transitioned from a gammaretrovirus to a third-generation lentiviral expression vector and removed epitope tags used to discriminate between CAR and CCR expression. Most strikingly, we found that inactivation of immune tyrosine activation motif 2 and 3 within our pCAR prototype markedly potentiated efficacy in xenograft-bearing NSG mice. Mechanistically, this resulted from increased pCAR T-cell functional persistence and organ infiltration, with enhanced local clearance of malignant B-cells. These data set the scene for evaluation of this iteratively honed pCAR candidate in a clinical trial in relapsed/refractory B-cell non-Hodgkin's lymphoma.

## Teaser

Here, we describe the late-stage optimisation of an avidity optimised CD19 parallel CAR for Phase 1 clinical evaluation. To minimise risk of insertional mutagenesis and immunogenicity respectively, we transitioned from a gammaretrovirus to a third generation lentiviral expression vector and removed epitope tags used to discriminate between CAR and CCR expression. Most strikingly, we found that inactivation of immune tyrosine activation motif 2 and 3 within our pCAR prototype markedly potentiated efficacy in xenograft-bearing NSG mice.

## Graphical Abstract

**Graphical Abstract:**
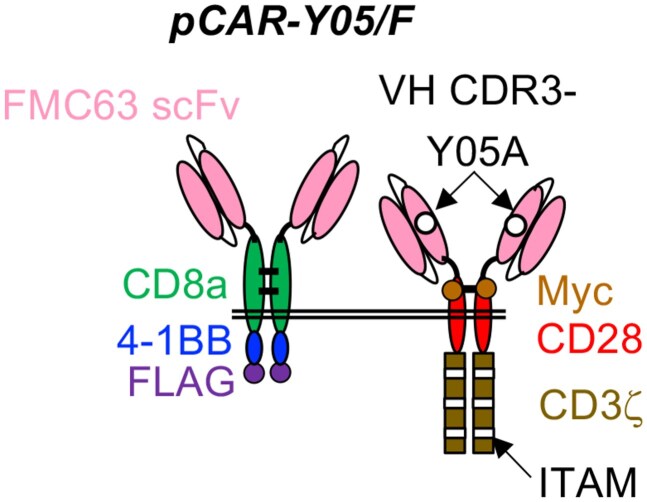

## Introduction

Chimeric antigen receptors (CARs) are synthetic fusion proteins that enable T-cells to engage and eliminate targets that express a cognate native cell surface antigen. The most effective and widely used CAR T-cell products are directed against the lineage-specific antigen, CD19, and have achieved profound efficacy in subjects with a variety of B-cell malignancies [1]. Clinical impact of CAR T-cells has been driven by the use of a second-generation (2G) design, originally conceived by Finney *et al*., in which a single CD28 or 4-1BB co-stimulatory module was incorporated upstream of an activating CD3ζ endodomain [2, 3]. Nonetheless, many subjects treated with 2G CAR T-cells relapse, an event that is commonly linked to inadequate CAR T-cell persistence [4, 5]. To address this, we designed the parallel (p)CAR system in which a 2G CAR is co-expressed with a chimeric co-stimulatory receptor (CCR) that provides a distinct second signal [6, 7]. Dual co-stimulation by CD28 and tumour necrosis factor receptors, such as 4-1BB, leads to amplified T-cell responses [8–10]. In keeping with this, we observed that CD28 + 4-1BB-based pCAR T-cells demonstrated improved metabolic fitness, enhanced anti-tumour efficacy, and sustained functional persistence in several models representative of a range of cancers [6, 7, 11]. Importantly, superior performance of pCAR-engineered T-cells has been independently described by several other research groups [12–16].

**Figure 1:**
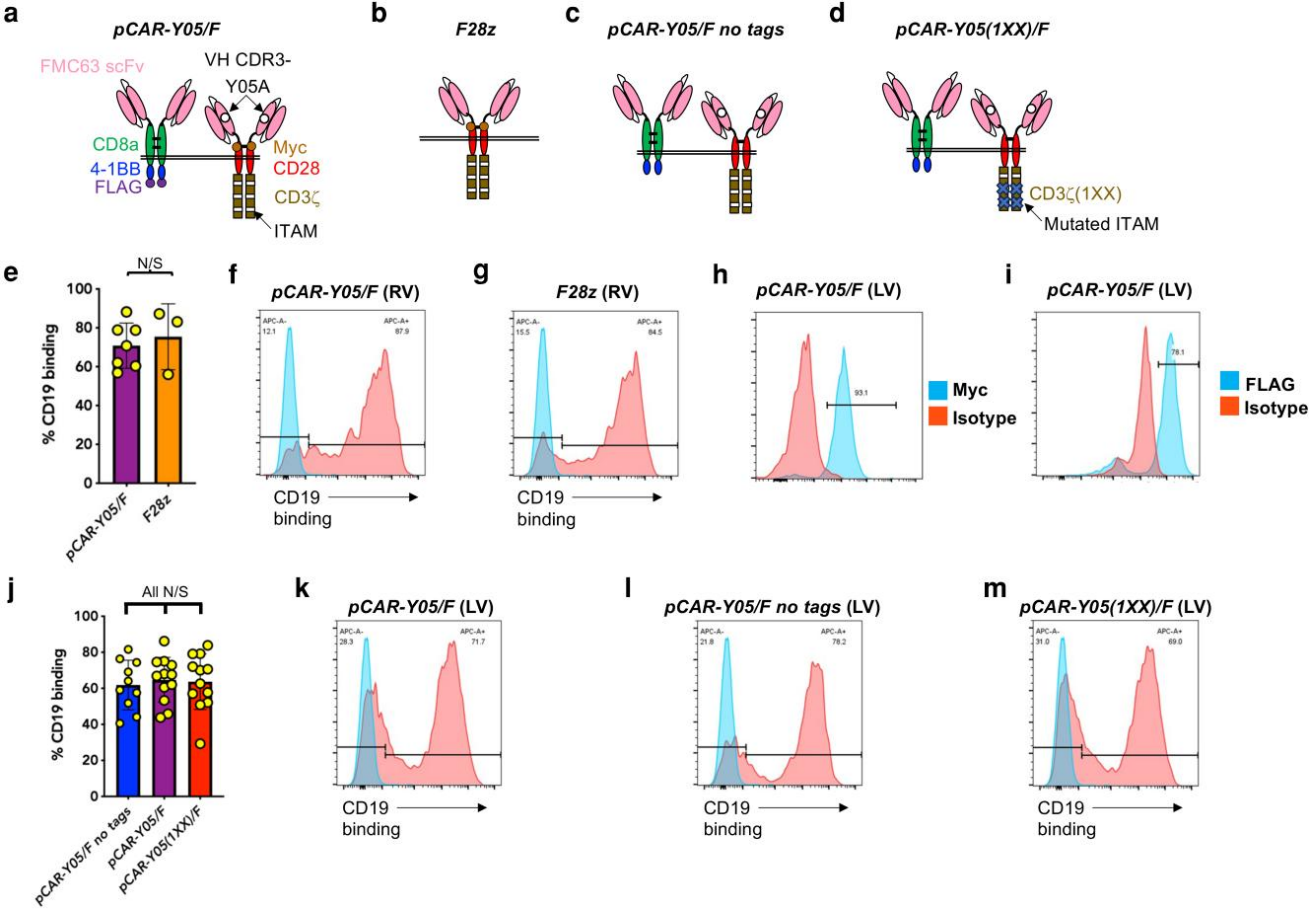
Structure and expression of CD19-targeted pCARs. Cartoon structures illustrating the structure of: (**a**) the parallel CAR, *pCAR-Y05/F*, (**b**) the 2G CAR *F28z,* (**c**) the parallel CARs *pCAR-Y05/F no tags,* and (**d**) *pCAR-Y05(1XX)/F*. Nomenclature of pCARs derives from a listing of the CAR binding moiety, followed by a forward slash and then the CCR binding moiety. (**e**) Transduction efficiency of human T-cells using the indicated SFG retroviral constructs, as determined by CD19-Fc binding (mean ± SD). N/S—not significant. Representative examples of transduction with (f) SFG *pCAR-Y05/F* and (**g**) SFG *F28z*. Jurkat cells were transduced with lentiviral-encoded *pCAR-Y05/F* and stained with (**h**) a 9e10 myc-specific antibody or, following permeabilization of the cells, (**i**) with a FLAG-specific antibody. Histograms show representative staining of the cells, making comparison with an isotype control. Similar results were obtained in three biological replicates. (**j**) Transduction efficiency of human T-cells using the indicated pVL lentiviral constructs (mean ± SD). N/S—not significant. Representative examples of transduction with (**k**) pVL *pCAR-Y05/F*, (**l**) pVL *pCAR-Y05/F no tags,* and (**m**) pVL *pCAR-Y05(1XX)/F*.

Recently, we adapted the pCAR platform to target CD19 by co-expressing a CD28-containing 2G CAR with a 4-1BB-containing CCR [11]. To direct CAR specificity, we employed an avidity-optimized derivative of a CD19-specific FMC63 scFv [17] in which the fifth tyrosine (Y05) present in complementarity determining region 3 of the variable heavy chain was replaced by alanine (A) [11]. Specificity of the accompanying CCR was directed using an unmodified FMC63 scFv. The structure of the resulting *pCAR-Y05/F* pCAR is shown in Fig. 1a. When evaluated in the widely used Nalm-6 xenograft model, treatment with *pCAR-Y05/F* T-cells achieved significantly greater efficacy than 2G CAR T-cells [11].

Given these encouraging results, we designed a Phase 1 clinical trial to test this technology in relapsed/refractory B-cell lymphoma. The objective of the present study was to maximally optimize the CD19 pCAR for clinical testing.

## Materials and methods

### CAR constructs

Constructs were initially expressed using the SFG gammaretroviral vector. The design of SFG *pCAR-Y05/F* (pCAR; Fig. 1a) and SFG *F28z* (2G control; Fig. 1b) has been described previously [11]. Stoichiometric co-expression of genes that encode CAR and CCR was achieved using a bicistronic vector in which an intervening *Porcine Teschovirus* (P2A) ribosomal skip peptide was placed downstream of an RRKR furin cleavage site and SGSG linker. The c-myc and FLAG epitope tags within SFG *pCAR-Y05/F* were removed by insertional mutagenesis to generate SFG *pCAR-Y05/F no tags* (Fig. 1c; Genscript, Leiden, The Netherlands). Subsequently, this pCAR construct was subjected to further insertional mutagenesis to introduce inactivating tyrosine (Y) -> phenylalanine (F) mutations in both ITAM 2 and 3 of CD3ζ to generate SFG *pCAR-Y05(1XX)/F* (Fig. 1d). Thereafter, each of these pCAR cDNAs was cloned using proprietary methodology into the pVL third-generation lentiviral vector by ViroCell Biologics, London, UK. The firefly luciferase/dsTomato red fluorescent protein (ffLuc/RFP) SFG retroviral vector used to modify Raji and Nalm-6 B-cell lines [18] and the CD19 vector used to modify LO68 cells have been previously described [11].

### Cell lines

Raji B-cell Burkitt's lymphoma, Jurkat acute T-cell leukaemia, and U937 myelomonocytic leukaemia cells were a gift from Dr Linda Barber, King's College London, London, UK. Ju77 and Ren mesothelioma cells were a gift from Prof Dean Fennell, University of Leicester, Leicester, UK. LO68 mesothelioma cells were a gift of Prof Tariq Sethi, King's College London, London, UK. A375 melanoma and both BxPC3 and CFPAC-1 pancreatic cancer cells were a gift of Prof John Marshall, Bart's Cancer Institute, Queen Mary University of London, London, UK. T47D and MDA-MB-468 breast cancer cells and MDA-MB-435 melanoma cells were gifts of Prof Joy Burchell, King's College London, London, UK. THP-1 myelomonocytic cells, A549 lung adenocarcinoma cells, and LS180 colon adenocarcinoma cells were purchased from the American Tissue Culture Collection (Manassas, VA). A2780 ovarian carcinoma cells were purchased from the European Collection of Authenticated Cell Cultures (Porton Down, UK). SKOV-3 ovarian carcinoma cells were a gift of Prof Sadaf Ghaem-Maghami, Imperial College London, London, UK. HN-3 head and neck cancer cells were a gift of Prof Suzanne Eccles, Institute of Cancer Research, Sutton, UK. Nalm-6 B-cell acute lymphoblastic leukaemia cells were a gift of Dr Robert Kochl, Francis Crick Institute, London, UK. All cell lines were validated by short tandem repeat analysis and were screened regularly for mycoplasma contamination by polymerase chain reaction.

### Transduction and expansion of human T-cells

#### Vector production

Retroviral vector was prepared by triple transfection of 293T cells (provided by the University College London Cancer Centre, London, UK). 293T cells (1.65 × 10^6^) were plated in 11 ml IMDM (ThermoFisher Scientific, Horsham, UK) + 10% FBS (Sigma-Aldrich, Poole, UK) in a 10 cm dish. The next day, GeneJuice (30 µl, Sigma-Aldrich) was added to 470 µl IMDM in an Eppendorf tube. After 5 min incubation at room temperature, 3.125 µg RD114 plasmid (gift of Prof M Collins, University College London, UK), 4.6875 µg pEQ-Pam3 plasmid (gift of Dr M Pule, University College London, UK), and 4.6875 µg SFG vector plasmid of interest (gift of Dr M Sadelain, Memorial Sloan Kettering Cancer Center, New York, NY) were added, mixed, and incubated for 15 min at room temperature. The mixture was then added slowly to the 293T cells and the plate was then incubated for 48 h in 5% CO_2_ in a 37°C incubator. Vector-containing medium was removed, snap frozen using an ethanol dry ice bath, and fresh medium was added to the plate. After a further 24 h, this procedure was repeated. Minibatches of pVL lentiviral vector encoding for all pCARs were generated and titrated by ViroCell Biologics using proprietary methods.

#### Human T-cell isolation, culture, and transduction

All work undertaken with human subjects was fully compliant with the Declaration of Helsinki. Healthy donors provided informed consent to participate in the study. Peripheral blood mononuclear cells (PBMC) were isolated from whole blood by Ficoll density gradient centrifugation under UK National Health Service Research Ethics Committee approval number 22/LO/0357. To facilitate transduction, PBMC were activated using TransAct (Miltenyi Biotec, Bergisch Gladbach, Germany), as recommended by the manufacturer. Retroviral transduction was conducted after 48 h on RetroNectin (Takara, Shiga, Japan)-coated non-tissue culture treated plasticware, as described [6]. Lentiviral transduction was similarly performed 48 h after PBMC activation but in the absence of RetroNectin, employing multiplicities of infection ranging from 10 to 100. Untransduced cells were added where necessary to normalize transduction efficiency, ensuring that a similar number of transduced cells were present in each condition. Activated PBMC and transduced T-cells were maintained in RPMI 1640 with L-glutamine (Lonza, Basel, Switzerland) + 5% human AB serum (Sigma-Aldrich; R5 medium) containing interleukin 2 (100 U/ml, Peprotec, London, UK).

### Flow cytometry

#### Assessment of T-cell transduction

All incubations were performed at 4°C for 30 minutes. Transduction efficiency was determined by incubation with soluble (s)CD19-Fc fusion protein (AcroBiosystems, Newark, NJ) followed by anti-human IgG Fc recombinant antibody—APC (BioLegend). Alternatively, 9e10 hybridoma supernatant was added (binds the myc epitope tag in some CARs), followed by goat anti-mouse IgG-PE conjugate (ThermoFisher Scientific). To detect CCR expression, intracellular staining was performed in Jurkat cells by fixation with 0.01% formaldehyde and permeabilization with PBS + 0.5% BSA + 0.1% saponin. Cells were stained with Anti-Flag tag—PE (L5) (BioLegend, San Diego, CA).

#### CD19 target specificity validation

To evaluate the target specificity of the FMC63 and FMC63 (Y05A) scFv, each was fused to human IgG1 Fc and expressed in Chinese Hamster Ovary cells using proprietary methods (Genscript). Binding to adherent cell lines was assessed after detachment with cell dissociation buffer (Gibco, UK) for 10 minutes at 37°C, followed by neutralization with DMEM (Gibco, UK) + 10% FBS (Sigma-Aldrich, Poole, UK). After sedimentation, pellets were re-suspended in fresh DMEM + 10% FBS and rested for 30 minutes at 37°C. All subsequent incubations were for 25 minutes at 4°C. Cells (10^5^ per test) were mixed with 1 µg/ml of each scFv-Fc fusion or an IgG1 Fc only control (Genscript), followed by secondary anti-human IgG Fc AF647 (BioLegend, San Diego, CA). For suspension cell lines, 10^5^ cells per test were pre-incubated with anti-human CD16, CD32, and CD64 (BioLegend, San Diego, CA) to block Fc receptors, followed by staining as described above.

#### Analysis of mouse tissues

All incubations were performed for 20 minutes at room temperature. Absolute numbers of CAR T-cells in blood were determined by flow cytometry with CountBright^TM^ absolute counting beads (50 µl per tube, containing 52 000 beads). Samples were stained with antibodies (all from BioLegend, unless indicated) directed against mouse CD45—APC/CY7, human CD45-BV510, human CD3—FITC, and sCD19-Fc (AcroBiosystems) followed by anti-human Fc-APC. CAR T-cells were defined as CD3^+^ sCD19-Fc^+^ events within a human CD45 gate. All samples were run until 5000 bead events were collected. Flow cytometry gating strategy used in whole blood is illustrated in Supplementary Figure S1. Cell count per µl was given using the following formula:


$$\#\;\;\mathrm{CA}R^{+\;}\;\mathrm{cells}/5000\times 52\;000/\mathrm{blood}\;\mathrm{volume}\;\left(\mu L\right)$$

Mouse tissues were de-aggregated using the gentleMACS^TM^ Octo Dissociator and stained with antibodies (all from BioLegend) targeted against mouse CD45-APC/CY7, human CD45-BV510, human CD3-APC, and human CD19-BV421.

#### Flow cytometry analysis

This was performed using a Beckman Coulter Cytoflex cytometer and data analysed using FlowJo version 9 (FlowJo, Ashland, OR). All gates were set using isotype control antibodies or fluorescence minus one controls.

### Cytotoxicity assays

Tumour cells were incubated with T-cells at a 1:1 effector to target (E:T) ratio for 72 h. For adherent cells, target cell viability was determined using an MTT assay as described [7]. Alternatively, for suspension cell targets, tumour cell viability was monitored using luciferase assays. D-luciferin (150 mg/ml, Cambridge Bioscience, Cambridge, UK) was added immediately before luminescence reading. In both cases, tumour cell viability was calculated using the following equation:


$$\begin{array}{l} {}[\mathrm{absorbance}\;\mathrm{or}\;\mathrm{luminescence}\;\mathrm{of}\;\mathrm{tumour}\;\mathrm{cells}\\ \;\mathrm{cultured}\;\mathrm{with}\;T\;\mathrm{cells}/\mathrm{absorbance}\;\mathrm{or}\;\mathrm{luminescence}\\ \mathrm{of}\;\mathrm{untreated}\;\mathrm{tumour}\;\mathrm{cells}\;\mathrm{alone}]\times 100\text{\%}. \end{array}$$

### Tumour re-stimulation assays

To compare *in vitro* activity, T-cells engineered to express the pCARs shown in Fig. 1 were iteratively co-cultured twice weekly with tumour cell lines, an assay which provides a convenient model of induction of CAR T-cell exhaustion [19]. T-cells were cultured with Raji or Nalm-6 tumour cells at a 1:1 E:T ratio in the absence of exogenous cytokine support. After 72 hours, supernatants were harvested and an equal number of fresh tumour cells added to re-stimulate the culture. Upon completion of each re-stimulation cycle, tumour viability was measured using a bioluminescence assay. A re-stimulation cycle was considered successful if viability was below 60%. Maximum fold T-cell expansion was calculated as the maximum ratio between the T-cell number at the end of a re-stimulation cycle divided by the starting T-cell number.

### Enzyme-linked immunosorbent assay

Supernatants were analysed using a human interferon (IFN)-γ enzyme-linked immunosorbent assay as described by the manufacturers (ThermoFisher Scientific).

### Xenograft studies

All animal experimentation was performed according to UK Home Office guidelines, as set out in project licence number P23115EBF. This work has also been approved by the King's College London animal welfare and ethical review body. NOD severe combined immunodeficiency gamma null (NSG) mice aged 6–10 weeks (both male and female) were purchased from Charles River Laboratories (Margate, UK). Mice were maintained at 20–24°C under enhanced SPF barrier conditions in individually ventilated cages on a 12:12 h light–dark cycle, with autoclaved bedding, irradiated chow, and sterilized water provided *ad libitum*. Sterilized environmental enrichment was provided, including nesting material, shelters, and simple tunnels. Animals were allowed to acclimatize for at least 1 week prior to experimental work.

Raji or Nalm-6 cells that expressed ffLuc/RFP were inoculated by intravenous (i.v.) injection in male and female NSG mice. Disease was allowed to establish for the indicated interval prior to pCAR T-cell treatment, which was also administered i.v., making comparison with PBS as a control. No specific criteria were used to include or exclude animals that were otherwise deemed fit for treatment. Mice with similar tumour-derived bioluminescence emission were randomly allocated to experimental or control groups, and treatments were administered in a randomized order (both generated using in house spreadsheet, which also randomized for cage number). PBS rather than untransduced or control transduced T-cells was selected as the control intervention since our focus was to compare the activity of the individual pCARs under study. To monitor disease burden, bioluminescence imaging was undertaken 2–3 times weekly. The experimental unit was single animals, analysed within groups that were treated similarly. No animals were excluded from the analysis except where tissue collection/processing was technically unacceptable. Experimental workers were blinded to the treatments allocated to each group. An independent scientist was aware of the randomization key at all stages of each experiment. Sample size was determined based on previously conducted experiments in similar models, using related CAR T-cell populations [11]. Expected adverse effects were transient weight loss and reduced mobility due to cytokine release syndrome, which was mitigated by the provision of a mushy diet. Primary outcomes measured were tumour-derived bioluminescence and survival (subject to endpoints listed below). Animals were subjected to regular clinical assessment and weighing and were humanely killed using a Schedule 1 procedure when (i) a durable and statistically significant difference in tumour burden between test and control groups was seen in at least three sequential bioluminescence measurements or (ii) if normal behaviour was limited without resolution within 48 hours, or in the event of loss of >15% body weight or major organ or behavioural dysfunction (e.g. jaundice, abdominal distension, dyspnoea, neurological signs, lameness, or general signs of ill-health such as piloerection, hunched posture, inability to groom, inactivity or inappetence).

### Statistical analysis

All data are derived from biological and technical replicates as specified in individual figure legends and were subjected to normality testing. Data analysis was performed using GraphPad Prism version 10.4. For analysis of data derived from multiple groups, one-way or two-way ANOVA was used (depending on the number of independent variables), followed by Tukey's multiple comparisons test. For non-parametrically distributed data, the Kruskal–Wallis test was used. Survival data were analysed using a Log rank (Mantel-Cox) test. When only two groups were compared, a Student's *t*-test or Mann–Whitney test was performed, depending on normality of the data. Paired testing was used only when comparisons were made between precisely matched CAR T-cell cultures derived from the same patients.

To determine effect size in xenograft studies, bioluminescence values were log_10_-transformed prior to analysis. Between-group comparisons at pre-specified timepoints were performed using an unpaired *t-test* with Welch's correction. Effect sizes are reported as ratios of geometric means with 95% confidence intervals, obtained by back-transformation of log-scale estimates.

## Results

### Evaluation of binding properties of Y05A scFv

**Figure 2:**
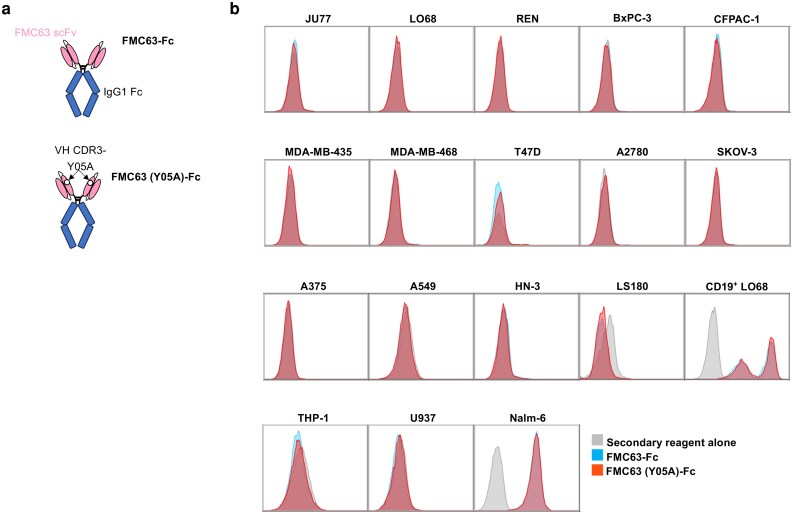
Binding studies undertaken using IgG1-scFv fusion proteins. **(a**) An unmodified FMC63 scFv or a derivative that contains a Y05A mutation in complementarity determining region (CRD) three of the FMC63 variable heavy (Vh) chain was fused to a human IgG1 Fc domain. Cartoon illustrations of the predicted structure are shown. (**b**) After expression in 293T cells, these fusion proteins were added to the indicated tumour cell lines in addition to CD19^+^ LO68 and Nalm-6 positive controls. Binding was detected by incubation with anti-human IgG1-Fc APC. Histograms indicate representative staining as detected by flow cytometry, making comparison with anti-human IgG1-Fc alone. Similar results were obtained in three biological replicates.

The CD19-specific FMC63 scFv has been extensively evaluated in the context of clinical CAR T-cell immunotherapy and no evidence of clinically significant off-target binding has emerged [20]. However, the introduction of the Y05A mutation into this scFv could conceivably have introduced a propensity for off-target engagement by this scFv. To exclude this, we first expressed the Y05A scFv as an Fc fusion protein, making a comparison with a similar fusion containing an unmodified FMC63 scFv (Fig. 2a) or IgG1 Fc control. All were evaluated for their ability to bind to a range of solid tumour cell lines derived from multiple tissue types. Reassuringly, no evidence of off-target binding by the FMC63 (Y05A) scFv-Fc fusion protein to any of these CD19-negative cell lines was observed, in contrast to both CD19-expressing positive controls (Fig. 2b).

### Functional evaluation of pCAR-Y05/F for off-target toxicity

**Figure 3:**
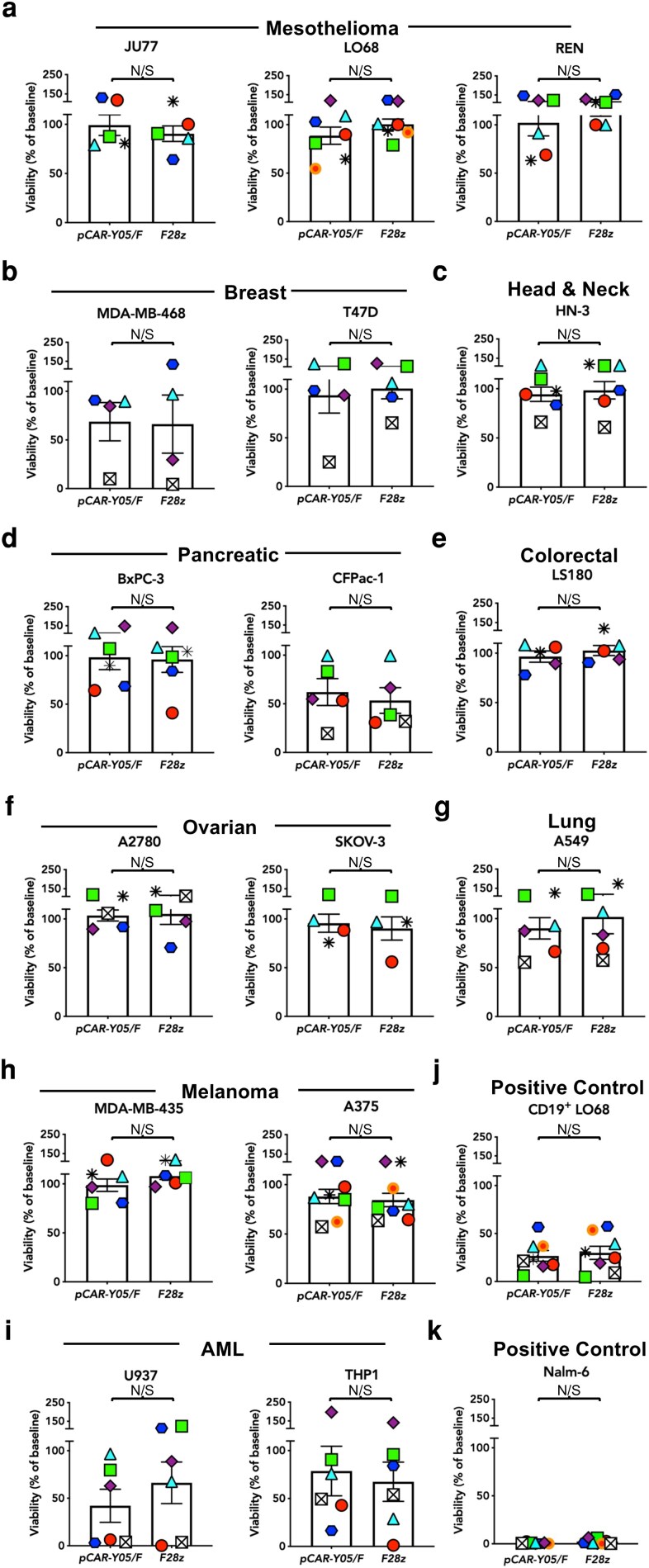
Comparison of on-target and off-target cytotoxic activity of *pCAR-Y05/F* with the second-generation *F28z* CAR. T-cells engineered to express *pCAR-Y05/F* or *F28z* were co-cultured for 72 hours at a 1:1 E:T ratio with the indicated (**a**) mesothelioma, (**b**) breast carcinoma, (**c**) squamous cell carcinoma of head and neck, (**d**) pancreatic carcinoma, (**e**) colorectal carcinoma, (**f**) ovarian carcinoma, (**g**) lung carcinoma, (**h**) melanoma, (**i**) AML, (**j**) CD19^+^ LO68 positive control or (**k**) Nalm-6 positive control. Target cell viability was assessed using MTT or a bioluminescence assay. Individual donors are indicated by different symbols/colours. Statistical analysis was by paired Student's *t*-test. N/S—not significant.

We next evaluated whether the Y05A mutation had unmasked off-target specificity in the context of pCAR T-cells. Activated PBMC were transduced with an SFG retroviral vector encoding *pCAR-Y05/F* and then co-cultivated with a range of solid tumour cell lines of diverse tissue origin, all of which lack CD19 when tested by flow cytometry and reverse transcriptase polymerase chain reaction (data not shown). Representative flow cytometry plots that demonstrate cell surface expression of both CAR and CCR encoded by SFG *pCAR-Y05/F* have been shown previously [11], exploiting the embedded myc and FLAG epitope tags within the CAR ectodomain and CCR endodomain, respectively (Fig. 1a). The cytotoxic activity of these T-cells against each tumour cell line was benchmarked against SFG-encoded *F28z* (Fig. 1b), which is an in-house produced analogue of the approved 2G product, axicabtagene ciloleucel. Both retroviral constructs achieved similar transduction efficiencies in human T-cells (Fig. 1e; representative plots shown in Fig. 1f and g, respectively). We observed that in the case of some sporadic donors, tumour cell viability was equally reduced when cultured with either CAR or pCAR T-cells. Possible explanations include antigen-independent cytotoxic activity of activated T-cells mediated via release of perforin/granzymes, cytokines (e.g. IFN-γ, TNF-α), expression of Fas ligand, TRAIL or NK receptors, enhanced adhesiveness, alloreactivity (especially with HLA class II-expressing leukaemic cells) and NK or γδ T-cell activity [21–23]. Importantly however, no significant killing or difference in tumour cell cytotoxicity was seen between *pCAR-Y05/F* and *F28z* T-cells when cultured with mesothelioma (Fig. 3a), breast cancer (Fig. 3b), head and neck squamous cell carcinoma (Fig. 3c), pancreatic cancer (Fig. 3d), colorectal cancer (Fig. 3e), ovarian cancer (Fig. 3f), lung cancer (Fig. 3g), melanoma (Fig. 3h), or acute myeloid leukaemia (AML) cell lines (Fig. 3i). By contrast, both T-cell populations were equally effective in mediating the killing of positive control cell lines, CD19^+^ LO68 (Fig. 3j) and Nalm-6 (Fig. 3k).

To confirm these findings, IFN-γ was measured in supernatants collected from these co-cultures after 72 hours. Although occasional donor-dependent increases were seen, most commonly in transduced T-cell cultures, once again, no differences were evident when comparing *pCAR-Y05/F* and *F28z* T-cells (Supplementary Figure S2). In summary, no evidence of off-target toxicity mediated by the inclusion of the Y05A-modified scFv in *pCAR-Y05/F* was uncovered in this analysis.

### Lentiviral expression of CD19-specific parallel CARs

Recently, a number of cases of T-cell malignancy were reported in patients treated with CAR T-cell immunotherapy [24]. Given the theoretical risk of insertional mutagenesis with the SFG gammaretroviral vector, which has long terminal repeat (LTR)-dependent enhancer activity [25], we elected to express all pCAR clinical candidates in T-cells using a third-generation lentiviral vector (pVL, ViroCell Biologics) that lacks LTR-derived enhancer function. To confirm that both chimeric receptors within *pCAR-Y05/F* were co-expressed using the pVL vector, we once again utilized the embedded myc and FLAG epitope tags to confirm CAR (Fig. 1h) and CCR expression (Fig. 1i) in transduced Jurkat cells using flow cytometry.

Removal of epitope tags is generally held to be desirable prior to clinical CAR T-cell evaluation since they may increase immunogenicity and/or influence binding properties and anti-tumour function [26], thereby impacting on product safety and efficacy. Consequently, we also generated an epitope tag-free pCAR variant, dubbed *pCAR-Y05/F no tags* (Fig. 1c). Additionally, we engineered a variant of the epitope tag-free pCAR in which ITAM 2 and 3 within the CD3ζ endodomain contained inactivating Y->F mutations (*pCAR-Y05(1XX)/F*, Fig. 1d). First described by Zhao *et al*. [27], the functional significance of the 1XX mutant endodomain was uncovered by Feucht *et al*. [28], who showed that it enhanced CAR T-cell anti-tumour activity. More recently, we found that a similar CD3ζ 1XX modification improved the function of an NKG2D-based adaptor CAR, yielding comparable function to a single ITAM Dap12-containing variant [29]. Lentiviral transduction efficiency of all constructs was similar (Fig. 1j; representative examples shown in Fig. 1k–m) and matched that earlier seen using the SFG retroviral vector (Fig. 1e).

### Comparison of *in vitro* anti-tumour function of CD19-specific parallel CARs

**Figure 4:**
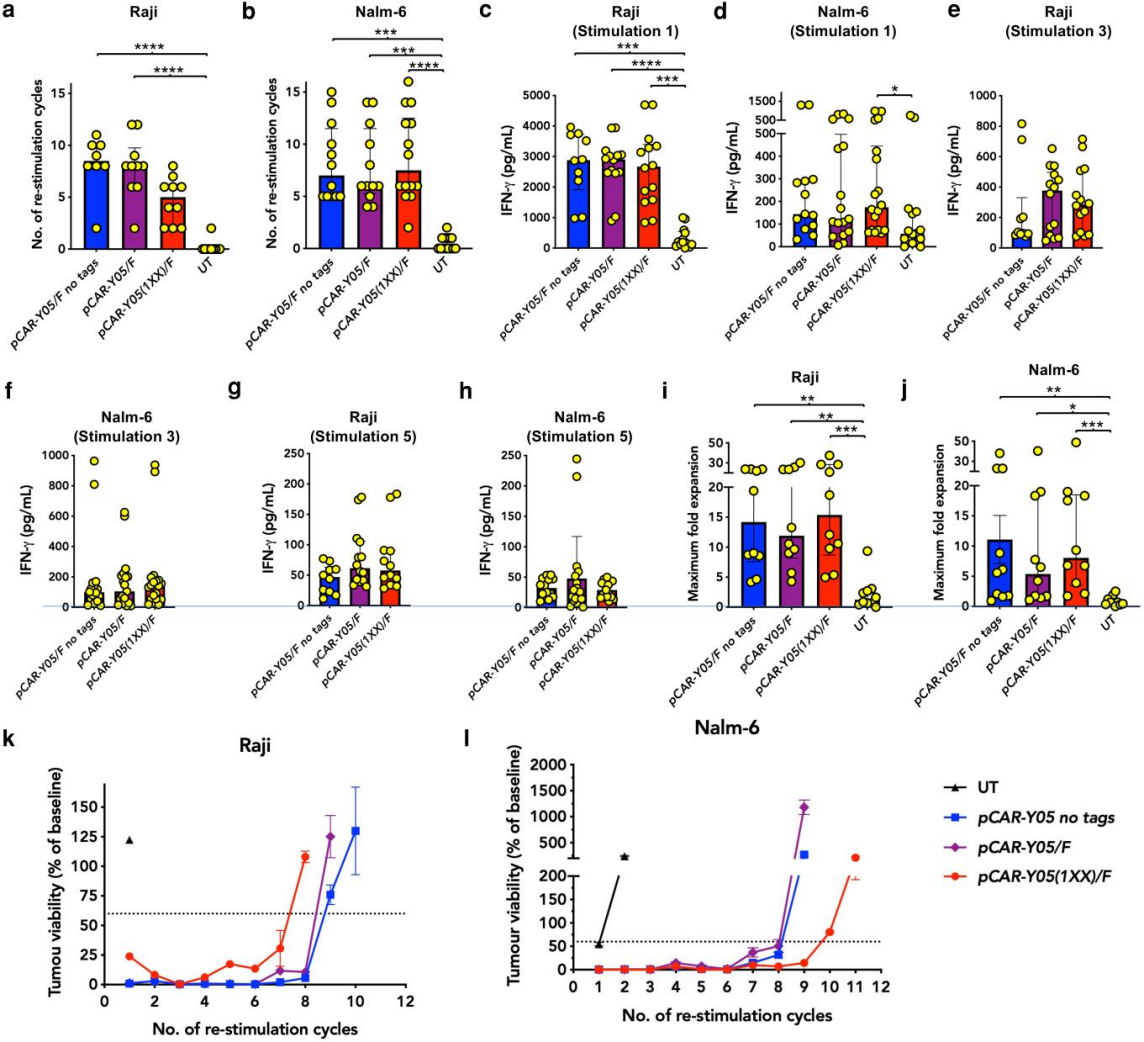
*In vitro* comparison of anti-tumour activity of CD19-specific pCAR candidates. Lentivirus-transduced T-cells that express the indicated pCARs were re-stimulated twice per week by co-culture with (**a**) Raji or (**b**) Nalm-6 tumour cells at a 1:1 effector to target ratio, making comparison with untransduced (UT) controls. Data show the median ± interquartile range of the number of re-stimulation cycles in which T-cells reduced residual tumour viability to below 60%. Supernatants were collected 72 hours after establishment of the first (Raji (**c**), Nalm-6 (**d**)), third (Raji (**e**), Nalm-6 (**f**)), and fifth re-stimulation cycle (Raji (**g**), Nalm-6 (**h**)). Data show median ± interquartile range of 1–2 technical replicates per donor from each of 5–10 independent donors. Maximum fold expansion of T-cells achieved during the re-stimulation cycles on (**i**) Raji and (**j**) Nalm-6, as described above (median ± interquartile range of 10 independent donors). All statistical analysis was by Kruskal–Wallis test. *P < 0.05, **P < 0.01, ***P < 0.001, ****P < 0.0001. Representative re-stimulation experiments on (**k**) Raji and (**l**) Nalm-6 in which tumour viability was determined at the end of each re-stimulation cycle using a bioluminescence assay (mean ± SEM, 2 technical replicates).

When repeatedly stimulated *in vitro* using Raji B-cell lymphoma (Fig. 4a) or Nalm-6 B-cell acute lymphoblastic leukaemia cells (Fig. 4b), all three lentiviral transduced pCAR T-cell populations underwent a similar number of productive re-stimulation cycles, defined as those in which residual tumour viability was <60%. Equivalent levels of IFN-γ were produced by these pCAR T-cell candidates after one (Fig. 4c and d), three (Fig. 4e and f), and five cycles of re-stimulation (Fig. 4g and h). Moreover, all pCARs achieved a comparable maximum fold T-cell expansion over these re-stimulation cycles (Fig. 4i and j). Notably, all of these assays revealed marked donor-dependent differences, most likely due to intrinsic differences in T-cell subset composition, differentiation state, and metabolic fitness. Consequently, all three pCAR candidates were advanced to *in vivo* testing to select a lead for Phase 1 clinical evaluation. Representative examples of tumour cell viability after each stimulation cycle are shown (Fig. 4k and l).

### Comparison of activity of CD19-specific parallel CARs against Raji lymphoma xenografts

**Figure 5:**
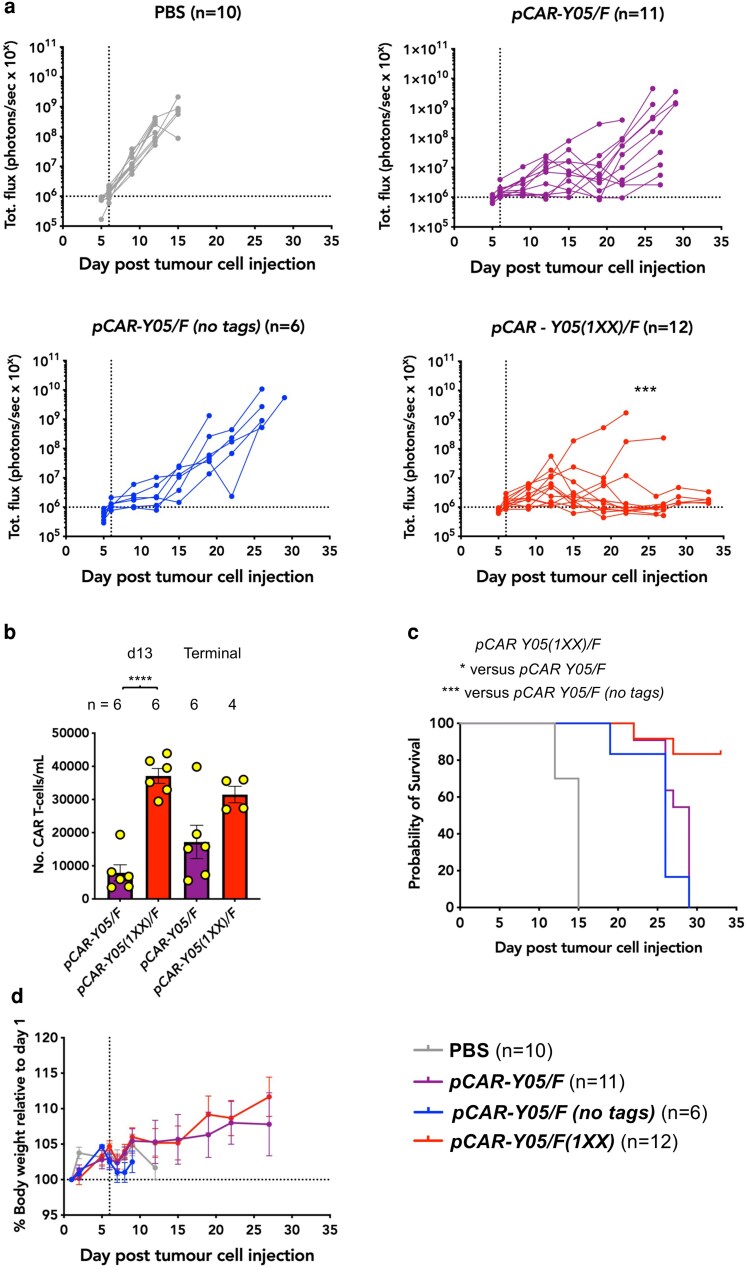
Evaluation of the efficacy of CD19 pCAR T-cells against Raji lymphoma xenografts. (**a**) NSG mice were inoculated i.v. on Day 1 with 1 × 10^5^ RFP/ffLuc^+^ Raji lymphoma cells. On Day 6 (vertical dotted line), 1 × 10^6^ of the indicated pCAR T-cell populations were infused i.v., making comparison with PBS (number of mice per group indicated). Tumour burden was monitored using serial bioluminescence imaging. Horizontal dotted line represents the limit of detection of tumour using bioluminescence. ****P < 0.0001, comparing *pCAR-Y05(1XX)/F* versus *pCAR-Y05/F* (+/− tags), on Day 26 using Welch's unpaired *t* test of log-transformed data. (**b**) Circulating CAR T-cells were enumerated by flow cytometry using TruCount on Day 13 and on the day of culling of mice (terminal). Statistical analysis was by an unpaired Student's *t*-test. ****P < 0.0001. (**c**) Kaplan–Meier survival curve. Statistical analysis was by the Log-rank (Mantel-Cox) test. *P < 0.05, ***P < 0.001. (**d**) Serial weight of mice in each group, relative to day of tumour inoculation (mean ± SEM).

Since we plan to clinically evaluate our CD19 pCAR technology in subjects with B-cell lymphoma, we next established a tumour model using ffLuc/RFP-expressing Raji lymphoma cells. Mice with six-day-established xenografts were treated i.v. with 1 × 10^6^ of the indicated pCAR T-cells or PBS as control. Figure 5a shows tumour-derived bioluminescence emission data from two pooled experiments involving a total of 39 mice. As expected, disease advanced very rapidly in PBS-treated mice, whereas progression was delayed in mice that received *pCAR-Y05/F* T-cells. In agreement with *in vitro* data presented in Fig. 4, the presence or absence of epitope tags in *pCAR-Y05/F* did not influence therapeutic efficacy, supporting their removal prior to clinical evaluation. Most strikingly, efficacy was significantly greater in mice treated with *pCAR-Y05(1XX)/F* CAR T-cells. In this group, disease burden remained below or close to the limit of detection in 10 of 12 mice (Fig. 5a). At Day 26, comparison of *pCAR-Y05(1XX)/F*–treated mice with the pooled pCAR control groups yielded a geometric mean ratio of 9.3 × 10^−4^ in bioluminescence emission (95% confidence intervals 3.5 × 10^−4^ to 2.5 × 10^−3^). This corresponds to an approximately 1000-fold reduction in tumour burden in *pCAR-Y05(1XX)/F*–treated mice. Absolute numbers of circulating CAR T-cells were also significantly higher when compared to those treated with *pCAR-Y05/F* T-cells (Fig. 5b). *pCAR-Y05(1XX)/F* T-cells also resulted in a significant increase in survival compared to either of the other pCAR groups (Fig. 5c). Treatment was not accompanied by weight loss in any case (Fig. 5d).

**Figure 6:**
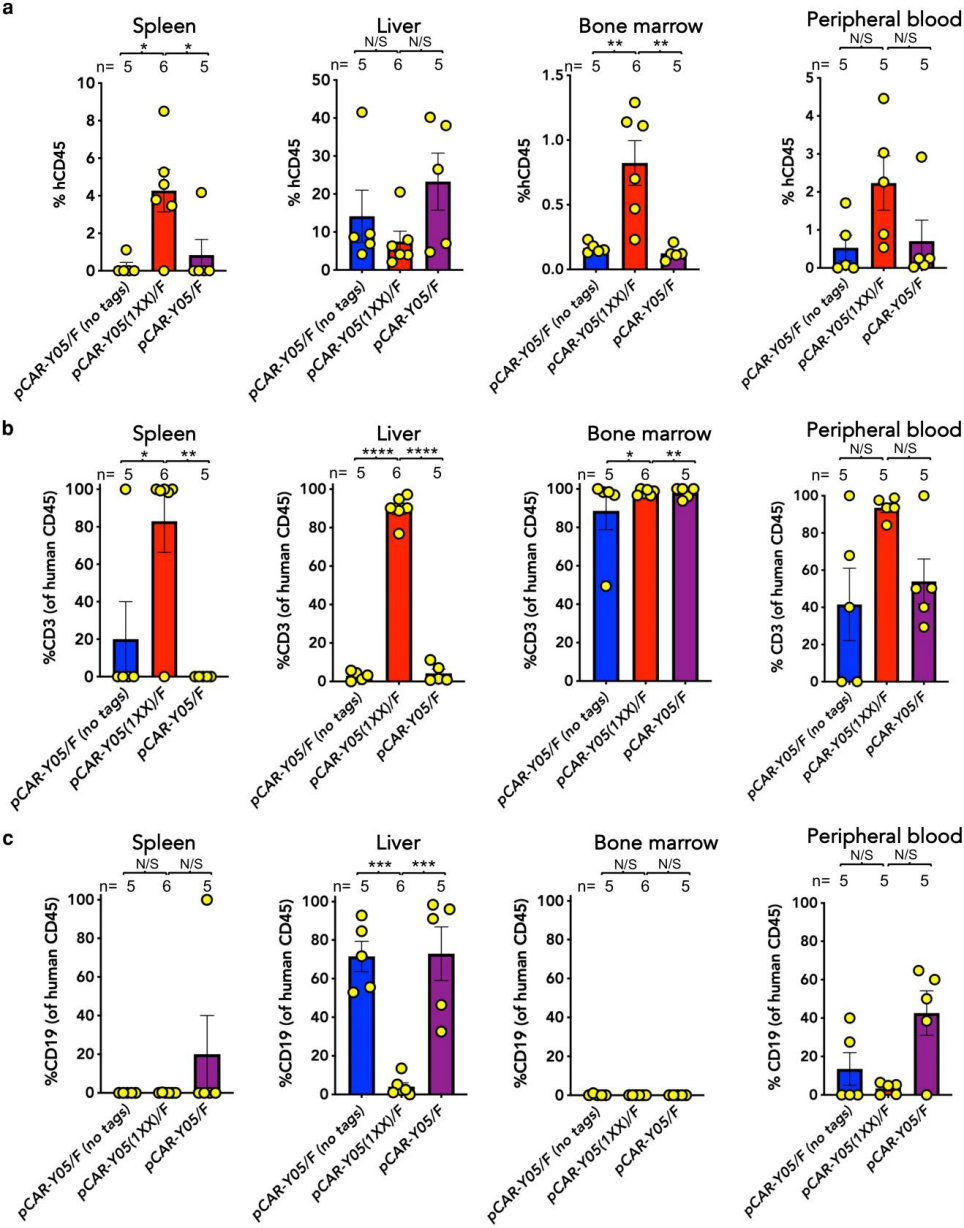
Analysis of pCAR T-cell and human B-cell distribution in Raji xenograft-bearing mice following treatment with the indicated pCAR T-cells. NSG mice were inoculated i.v. on Day 1 with 5 × 10^5^ RFP/ffLuc^+^ Raji lymphoma cells. On Day 6, 1 × 10^6^ of the indicated pCAR T-cell populations were infused i.v. mice were killed on Day 33, or earlier, if humane endpoints were met and spleen, liver, and bone marrow were harvested and analysed by flow cytometry for (**a**) percentage human CD45^+^ cells (number of mice per group indicated). Within a human CD45 gate, (**b**) percentage human T-cells (CD3^+^), and (**c**) percentage human B-cells (CD19^+^) were determined. The number of mice is indicated above each panel. Statistical analysis was performed by one-way ANOVA. *P < 0.05, **P < 0.01, ***P < 0.001, ****P < 0.0001, N/S—not significant.

Next, we sought to understand mechanisms underlying these functional differences. Terminally harvested spleen, liver, and bone marrow were analysed for percentage human CD45^+^ cells (Fig. 6a) within which we determined the proportions of T-cells (representative of CAR T-cells, Fig. 6b) and B-cells (indicative of Raji tumour cells, Fig. 6c). The human CD45% was significantly higher in spleen, bone marrow, and peripheral blood of *pCAR-Y05(1XX)/F* CAR T-cell treated mice, although differences in liver did not reach statistical significance (Fig. 6a). Percentage of T-cells was significantly higher in the spleen and—most strikingly—the liver (Fig. 6b) of mice following *pCAR-Y05(1XX)/F* CAR T-cell treatment. The importance of this finding stems from the fact that liver was the main site of CAR T-cell treatment failure, indicated by significantly higher percentages of hepatic CD19^+^ cells in *pCAR-Y05/F-* and *pCAR-Y05/F (no tags)-*treated mice (Fig. 6c). No differences in bone marrow T-cell percentage were observed between treatment groups (Fig. 6b), while B-cells were not detected at this site in any of these groups (Fig. 6c).

### Comparison of activity of CD19-specific parallel CARs against Nalm-6 leukaemic xenografts

**Figure 7:**
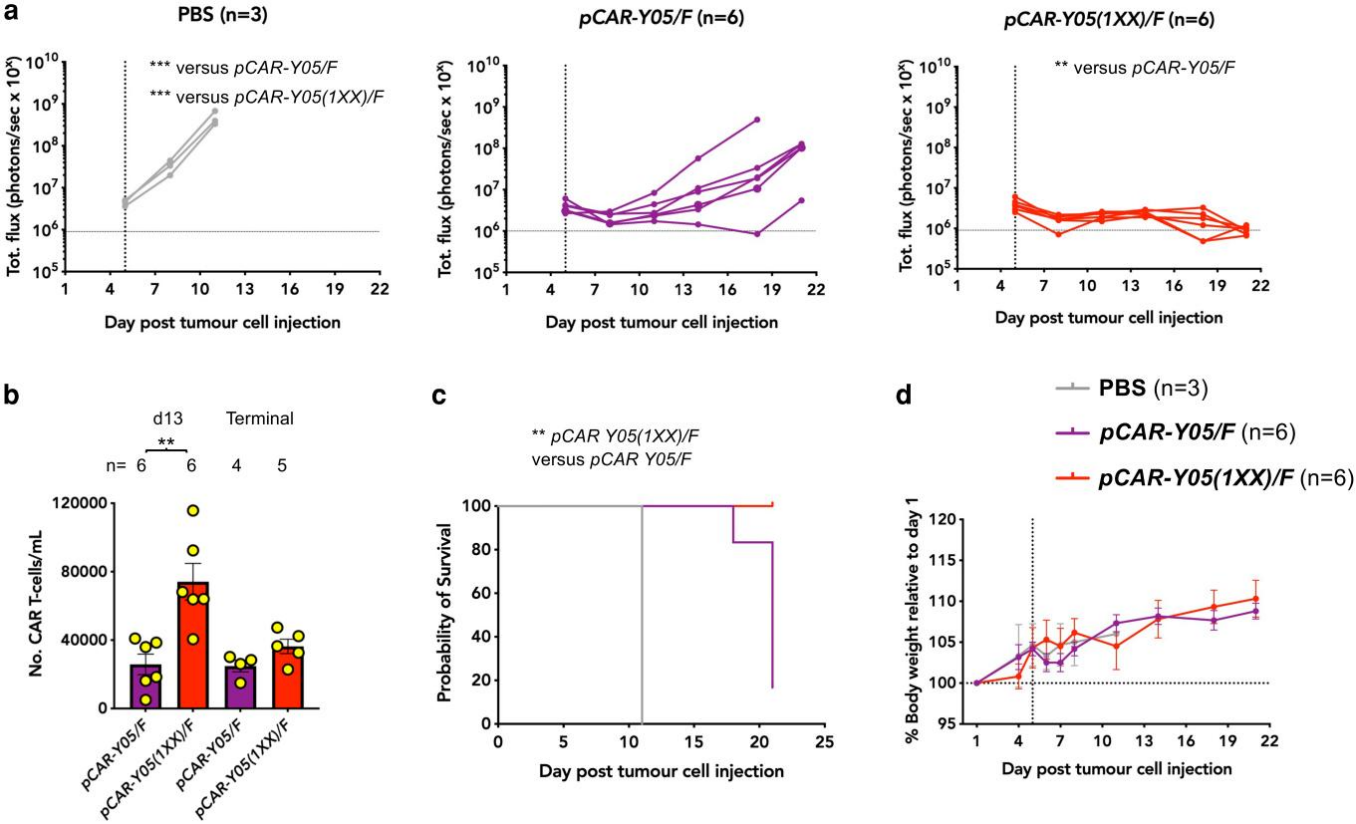
Evaluation of the efficacy of CD19 pCAR T-cells against Nalm-6 B-ALL xenografts. (**a**) NSG mice were inoculated i.v. on Day 1 with 5 × 10^5^ RFP/ffLuc^+^ Nalm-6 cells. On Day 5 (vertical dotted line), 1 × 10^6^ of the indicated pCAR T-cell populations were infused i.v., making comparison with PBS (number of mice per group indicated). Tumour burden following treatment was monitored by serial bioluminescence imaging. A horizontal dotted line represents the limit of detection of tumour using bioluminescence. ***P < 0.01, comparing PBS with either pCAR post treatment by two-way ANOVA; **P < 0.01, comparing *pCAR-Y05(1XX)/F* versus *pCAR-Y05/F*, on Day 21 using Welch's unpaired *t* test of log transformed data. (**b**) Circulating CAR T-cells were enumerated by flow cytometry using TruCount on Day 13 and on the day of culling of mice (terminal). Statistical analysis was by an unpaired Student's *t*-test. **P < 0.01. (**c**) Kaplan–Meier survival curve. Statistical analysis was by the Log-rank (Mantel-Cox) test. **P < 0.01. (**d**) Serial weight of mice in each group, relative to day of tumour inoculation (mean ± SEM).

Next, a similar experiment was conducted in mice with a Nalm-6 xenograft, comparing *pCAR-Y05(1XX)/F* with the originally described CD19-specific pCAR, *pCAR-Y05/F.* On Day 1, 0.5 × 10^6^ Nalm-6 cells were administered i.v. in NSG mice. On Day 5, mice received 1 × 10^6^ pCAR T-cells i.v., or PBS as control (*n* = 15 mice in total). The PBS control group was limited to three animals in accordance with the principle of Reduction under the 3Rs [30], as tumour growth in control mice in this model is highly consistent and predictable [11]. Consequently, a small cohort was considered sufficient by the investigating team to confirm the expected control trajectory while avoiding unnecessary animal use. Although the small PBS group rendered the study underpowered in the strict sense, Fig. 7a shows that rapid and uniform disease progression occurred in all three of these mice and this was significantly delayed following treatment with either pCAR. Moreover, mice treated with *pCAR-Y05(1XX)/F* T-cells achieved significantly improved efficacy compared to *pCAR-Y05/F* (Fig. 7a), which was at or close to the limit of disease detection in all six mice. At Day 21, comparison between *pCAR-Y05(1XX)/F*− and *pCAR-Y05/F*–treated mice yielded a geometric mean ratio of 0.014 (95% confidence intervals 0.0027–0.078), corresponding to an approximately 70-fold reduction in tumour burden. As before, this was accompanied by elevated levels of circulating CAR T-cells (Fig. 7b) and increased survival in the *pCAR-Y05(1XX)/F* treated group (Fig. 7c). Treatment was not accompanied by weight loss in any case (Fig. 7d). Together, these data strongly support selection of the *Y05(1XX)/F pCAR* candidate for first-in-human evaluation.

## Discussion

We have previously reported that CAR T-cell immunotherapy can be potentiated through the use of a pCAR architecture in which a 2G CAR is co-expressed with a CCR that provides a distinct co-stimulatory signal [6, 7]. To apply this to treat CD19^+^ B-cell malignancy, we co-expressed an avidity-optimized CD28 CAR (containing a Y05A mutation in complementarity determining region 3 of the FMC63 scFv variable heavy chain) with a 4-1BB-based CCR targeted using an unmodified FMC63 scFv. Having confirmed its superior efficacy *in vivo* [11], we next transitioned this pCAR approach for Phase 1 clinical evaluation in patients with relapsed/refractory B-cell non-Hodgkin's lymphoma. Owing to the inclusion of a modified FMC63 scFv to direct CAR specificity, we first confirmed that the resulting pCAR did not engage in off-target binding or activity. Next, we switched from a gammaretroviral vector to a third-generation lentiviral vector, which is a preferred expression system to reduce the risk of insertional mutagenesis. We also confirmed that no detrimental effects ensued following the removal of myc and FLAG epitope tags, which had originally been included to discriminate between CAR and CCR components of the pCAR, respectively.

The most striking finding of our study was the profound increase in therapeutic efficacy observed when the pCAR architecture was modified to include a 1XX CD3ζ endodomain in which ITAM 2 and 3 were inactivated by mutation. This finding was reproducible in two separate xenograft models conducted using T-cells from two independent donors. Previously, it has been shown that inclusion of a 1XX CD3ζ domain in an otherwise conventional 2G CAR resulted in increased T-cell persistence accompanied by reduced differentiation and exhaustion marker expression [28]. We also found that the circulating CAR T-cell number was increased when pCAR T-cells incorporated a 1XX endodomain. More recently, this modification was also found to potentiate solid tumour-targeted CAR T-cells [31]. Once again, retarded differentiation and enhanced persistence of CAR T-cells were observed. However, rather than downregulating signalling, investigators reported that this attenuated endodomain resulted in increased calcium flux and more rapid activation of the mitogen-activated protein kinase pathway, when compared to a counterpart containing three intact ITAMs [31].

A 1XX endodomain containing pCAR has recently been described that co-targeted ADGRE2 and CLEC12 in order to provide a therapeutic approach for AML [16]. The focus of that study was to employ the pCAR architecture to the stringency of discrimination between normal and malignant cells, owing to their differential expression of this target antigen combination [6]. However, comparison was not made with a triple ITAM-intact control and thus mechanisms by which the 1XX endodomain may have boosted function remained unexplored in the pCAR (as opposed to 2G CAR) context.

We uncovered a previously undescribed mechanism by which the 1XX CD3ζ domain boosts the anti-cancer activity of pCAR-engineered T-cells. Specifically, we observed a profound change in the distribution of disease in Raji-engrafted mice treated with the various CD19-specific pCARs under study. All pCAR T-cells proved effective in reducing or eradicating disease located in bone marrow—a site at which these engineered T-cells proved plentiful. However, treatment with both *pCAR-Y05/F* and *pCAR-Y05/F (no tags)* T-cells did not prevent disease progression in the liver, ultimately accounting for therapeutic failure. By contrast, hepatic disease burden was efficiently controlled in mice that received *pCAR-Y05(1XX)/F* T-cells, accompanied by a significant increase in the percentage of these T-cells at that location.

It is widely recognized that the liver represents a highly tolerogenic organ, perhaps of necessity owing to its exposure to large quantities of gut-derived exogenous antigen [32, 33]. In keeping with this, hepatic allografts are significantly less prone to immune-mediated rejection than other solid organ transplants, meaning that a sizeable minority of patients can be weaned from immunosuppression [34]. Moreover, in solid tumour CAR T-cell immunotherapy, increased numbers of hepatic myeloid-derived suppressor cells have been reported to compromise CAR T-cell activity at that site [35]. The additional therapeutic challenge imposed by extramedullary disease has also been highlighted in both CD19-expressing malignancy [36] and in AML [37]. Together, our data argue that the optimal balance of activating and co-stimulatory signals delivered by the *Y05(1XX)/F* pCAR enabled adoptively infused T-cells not only to maintain enhanced functional persistence in the circulation (Figs. 5b and 7b) but also to access and expand within diseased organs including the liver (Fig. 6b), effectively and uniquely clearing disease at that site (Fig. 6c).

An important question raised by our study is why ITAM attenuation in the *Y05(1XX)/F* pCAR confers a profound *in vivo* advantage despite no detectable *in vitro* difference?

Possible explanations include the fact that *in vivo* activity requires persistence and trafficking to diseased sites (notably liver) that impose a locally immunosuppressive microenvironment. Additionally, tonic signalling differences that only manifest under conditions of limiting antigen exposure may have favoured the activity of the 1XX pCAR. Moreover, the lack of any observable difference in IFN-γ production and proliferation across multiple stimulation cycles (given also the number of donors tested) calls into question how predictive such studies are of performance in a living host.

Our study has a number of important limitations. While superior anti-tumour activity of the CD19 pCAR platform over a matched 2G design was demonstrated previously [11], this comparison has not been undertaken with a third-generation (3G) CAR. However, pCAR T-cells targeted against either MUC1 or colony-stimulating factor all outperformed matched 3G CAR T-cells in previous studies [6]. In that work, it was also shown that 3G CARs deliver sub-optimal co-stimulation owing to non-physiological positioning of the downstream co-stimulatory domain, a finding that may account for their lack of clinical impact. A second important limitation is that the mechanistic basis for the superior anti-tumour activity of the *Y05(1XX)/F* pCAR was not fully explored. While we did demonstrate an increase in circulating CAR T-cell numbers and greater hepatic T-cell infiltration with the 1XX pCAR, markers of exhaustion, memory phenotype, and proliferative capacity require further investigation in both circulating and intrahepatic CAR T-cells.

In conclusion, we describe the stepwise transition of an experimental CD19-specific T-cell therapy from the research laboratory to a Phase 1 clinic-ready drug product for patients with refractory B-cell lymphoma. Using an avidity-optimized scFv, we engineered a pCAR lacking epitope tags that delivers dual CD28 and 4-1BB co-stimulation and a fine-tuned activation signal. When expressed using a clinical stage lentiviral vector, the 1XX pCAR platform achieved markedly improved disease control owing to enhanced CAR T-cell expansion and efficient infiltration of organs, including the liver, a known site of immune privilege. Our study is limited by the lack of confirmation of these findings in immunocompetent model systems. To evaluate generalizability of these data, a Phase 1 evaluation of *pCAR-Y05(1XX)/F* T-cells in patients with relapsed refractory B-cell non-Hodgkin's lymphoma is at an advanced stage of preparation and is scheduled to commence in 2026.

## Supplementary Material

uxag032_Supplementary_Data

## Data Availability

The datasets used and/or analysed during the current study are available from the corresponding author on reasonable request.
