# Supplementary material for: Optimization of a parallel CAR for B-cell lymphoma via ITAM attenuation and target specificity validation
Source: Clin Exp Immunol. 2026 May 26;220(1):uxag032. doi: 10.1093/cei/uxag032 (PMC13242215; doi:10.1093/cei/uxag032)
Supplement: uxag032_Supplementary_Data [file uxag032_supplementary_data.docx]

**Supplementary material**

**
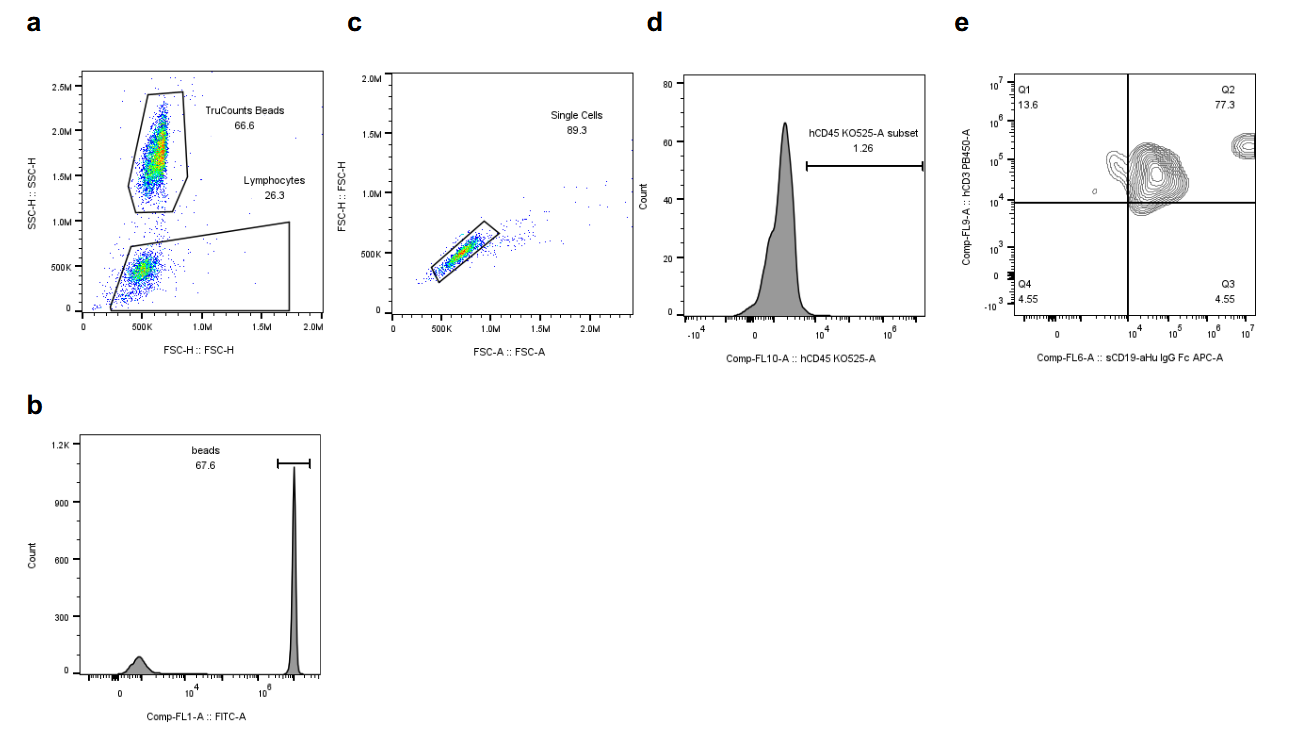
**

**Supplemental Fig. 1**

Gating strategy used for cell counting in blood. a Lymphocytes and counting beads were distinguished on a forward versus side scatter gate and beads displayed additionally as a histogram plot. b Within the lymphocyte gate, cells were sequentially gated on c singlets, d human CD45+ events and e CD3+ events that bound sCD19-Fc.


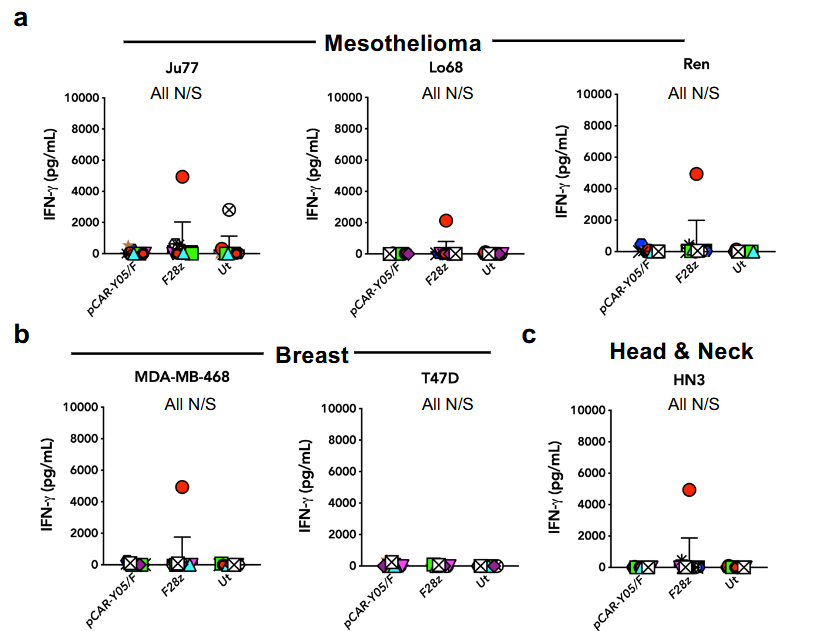

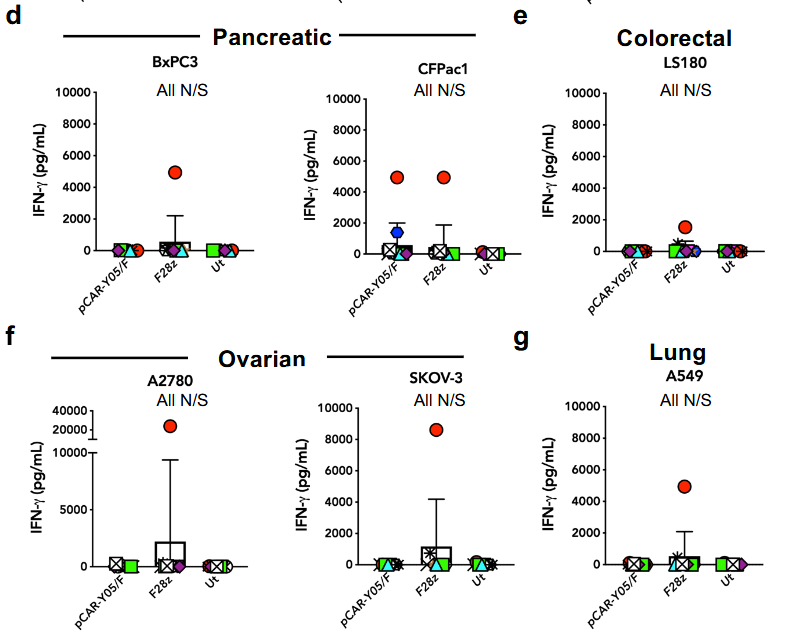

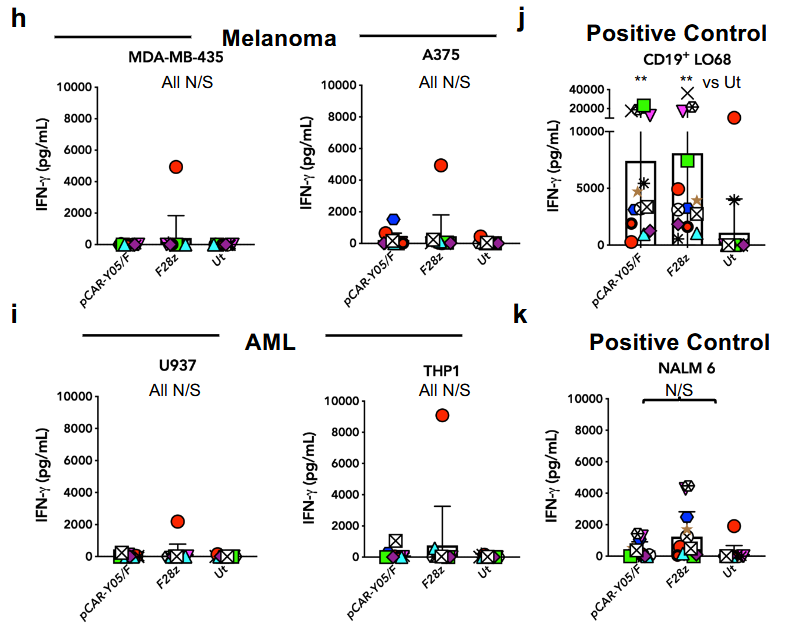
`

**Supplemental Fig. 2** Assessment of on-target and off-target IFN-g production by pCAR-Y05/F T-cells. T-cells engineered to express pCAR-Y05/F or F28z were co-cultured for 72 hours at a 1:1 effector to target ratio with the indicated a mesothelioma, b breast carcinoma, c squamous cell carcinoma of head and neck, d pancreatic carcinoma, e colorectal carcinoma, f ovarian carcinoma, g lung carcinoma, h melanoma, i AML, j CD19+ LO68 positive control or k Nalm-6 positive control. Concentration of IFN-g in harvested supernatant was determined by ELISA. Individual donors are indicated by different symbols/ colours. Statistical analysis was by one way ANOVA. N/S – not significant.
